# Simulated winter climate change reveals greater cold than warm temperature tolerance in *Chrysolina polita* (Coleoptera: Chrysomelidae)

**DOI:** 10.1093/ee/nvae120

**Published:** 2024-12-17

**Authors:** Anni Palvi, Leena Lindström, Aigi Margus

**Affiliations:** Department of Biological and Environmental Science, University of Jyväskylä, Jyväskylä, Finland; Department of Biological and Environmental Science, University of Jyväskylä, Jyväskylä, Finland; Department of Biological and Environmental Science, University of Jyväskylä, Jyväskylä, Finland

**Keywords:** adaptation, overwintering, metabolism, thermotolerance

## Abstract

Climate change is expected to lead to rising winter temperatures in temperate zones, coinciding with a decrease in winter snow cover. Insects adapted to winter conditions in the temperate zone might be exposed to changing winter conditions and higher temperature fluctuations, which can affect diapause and mortality. We studied the effects of climate change on *Chrysolina polita*, a temperate zone species overwintering as an adult in the shallow surface of the soil. We tested the effects of increased and fluctuating temperature on the mortality and body composition of the beetles in a laboratory environment, as well as the effects of snow cover removal on the mortality and body mass in field conditions. We found that in the laboratory study, a 2 °C increase in mean temperature increased mortality and resulted in increased lipid consumption, whereas temperature fluctuation caused desiccation of the beetles but did not affect mortality compared to the control condition. In the field study, the snow removal caused the mean soil temperature to decrease by 3 °C and fluctuate (ranging from −26.4 to 2.5 °C compared to a range of −1.7 to 0.5 °C in the control), yet these differences did not affect beetle mortality or body mass. We conclude that *C. polita* exhibits greater resistance to cold temperatures than to higher temperatures during diapause. Therefore, the rising temperatures associated with climate change can pose challenges for overwintering.

## Graphical Abstract

**Graphical Abstract:**
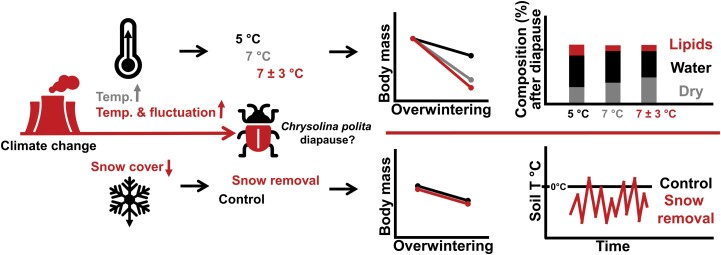

## Introduction

Climate change is predicted to have extreme effects on the winter conditions in the temperate zone. The global surface temperature is likely to rise at least 1.5 °C in the next decades regardless of the carbon dioxide emission -scenario, and, depending on the scenario, from 1.4 to 4.4 °C until the end of the century (IPCC 2021). Temperature increase in higher latitudes is predicted to be higher than the global average (Ruosteenoja and Jylhä 2021), and the winter temperature increase to be even more than summer (IPCC 2021, [Bibr CIT0024][Bibr CIT0036]). Rising global temperatures are expected to reduce the total area of winter snow cover, and according to various climate scenarios, a 1.5 °C increase in global temperature could lead to a 5% reduction in total area of the land under the snow cover while a more substantial 4 °C increase could decrease snow cover by up to 26% (IPCC 2021).

The snow acts as a buffer between air and soil, and according to Goncharova et al. (2019), a snow depth of 20 cm is needed for the buffering effect. As snow cover decreases, the ground might become more exposed to fluctuating air temperature (Goncharova et al. 2019), which can cause deeper soil freezing (Iwata et al. 2010). Consequently, soil temperatures may contrarily get colder due to climate change if the buffering effect of snow cover decreases (Iwata et al. 2010, Tan et al. 2014). On the other hand, rising air temperatures might offset the potential decrease in soil temperature. Additionally, decreasing albedo and increasing heat absorption by the ground (Zhang et al. 2008) may further prevent soil temperatures from dropping.

The complex interplay of different factors makes it challenging to predict how climate change is going to affect soil microclimate during the winter. Many temperate zone insect species overwinter in the soil, and higher winter temperatures can cause disturbance of diapause and increase the mortality rate for the freeze-tolerant overwintering insect species (Bale and Hayward 2010). For these species, the warmer overwintering conditions can lead to a higher metabolism and, thus, cause insects to lose more of their body mass content, causing higher mortality and other negative consequences (Irwin and Lee 2003, Sorvari et al. 2011, Vesterlund et al. 2014, Klockmann and Fischer 2019). In some species, apart from the cues from decreasing amount of light and nutrition quality (Danks 1987), the start of diapause also requires exposure to cold, and if the temperature is too high, the diapause maintenance period might be abandoned (Bradshaw and Holzapfel 2010). These effects can cause a significant decrease in the overwintering success of freeze-tolerant insect species due to climate change (Bale and Hayward 2010).

In this study, we studied the effects of the predicted temperature changes due to climate change on the knotgrass leaf beetle (*Chrysolina polita* L., Chrysomelidae: Coleoptera). These small, 6.5–8.5 mm size herbivores use *Mentha* genus and other Lamiaceae family species as host plants (Stork 1980). They overwinter in shallow soil surfaces (Lehmann et al. 2015) as an adult and can be mobile during the diapause. They follow a facultative diapause, so their diapause depends highly on environmental cues such as temperature and light (Daloze and Pasteels 1978). *C. polita* is a widespread and common species and according to observational data, it has spread widely all over Europe (iNaturalist 2023). These features make it a well-suited species for studying the effects of climate change on overwintering of insects in the temperate zone.

We tested 2 scenarios: (i) an increase in winter temperature under laboratory conditions and (ii) the effects of snow removal under field conditions. Climate change was assumed to cause a 2 °C increase in winter temperatures, and snowless conditions were further expected to lead to temperature fluctuations. We tested these conditions in a controlled laboratory experiment and asked how the 2 °C temperature increase, daily temperature fluctuation, and sex (female, male) affect mortality, body mass, and body composition of *C. polita*. Additionally, in the field experiment, we asked how snow removal affects soil temperature, beetle mortality and body mass.

## Materials and Methods

We collected the knotgrass leaf beetles (*C. polita*) during August and September 2020 from the arable land in Kortesuo-Viitaniemi, Jyväskylä, Finland (N 62.249188; E 25.727169). The beetles were brought to the University of Jyväskylä laboratory and first kept in 120 ml jars (Laplex), grouped by collection date, with each jar containing 10 beetles. These jars were equipped with net covers to ensure free air circulation, light penetration, and temperature flow. After completing the collection, we removed any dead or weak beetles to maintain robust groups of 10. For experimental grouping, a single beetle was randomly selected from each jar to create 3 laboratory groups of 46–50 and 4 field groups of 30 (Tables 1 and 2), ensuring a consistent representation from each collection date across all groups.

**Table 1.** The number of dead and survived *Chrysolina polita* beetles and the mortality (%) for females and males across different treatment groups in the laboratory experiment

Treatment	Sex	Dead	Survived	Mortality (%)
5 °C	Female	0	23	0.0
	Male	0	27	0.0
7 °C	Female	4	17	23.5
	Male	4	23	17.4
7 ± 3 °C	Female	0	23	0.0
	Male	4	19	21.1

**Table 2.** The before and after winter body masses (mg) and mortalities (dead/survived) of the *Chrysolina polita* for females and males across different treatments in the 4 treatment blocks of the field experiment

Treatment block	Sex	Before winter mass (mg)		After winter mass (mg)		Dead/Survived
		Mean	SD	Mean	SD	
Control 1	Female	65.4	3.4	60.4	3.4	0/14
	Male	43.7	4.2	40.1	4.0	1/15
Control 2	Female	61.8	7.1	60.2	6.1	0/14
	Male	41.0	4.4	39.2	5.2	0/16
Snow removal 1	Female	65.0	7.9	60.7	6.6	0/12
	Male	42.4	4.8	40.6	5.0	0/18
Snow removal 2	Female	63.4	4.1	60.5	4.3	0/14
	Male	42.4	4.1	40.7	5.2	3/13

At the start of the experiment, we weighed the beetles with Mettler Toledo (Switzerland) AM100 analytical balance and determined their sexes (female, male), after which we verified that there was no statistically significant difference in mass between the treatment groups (laboratory experiment: F_1,141_ = 0.34; *P* = 0.712; field experiment: *F*_1,118_ = 0.04, *P* = 0.849). In addition, we confirmed that the distribution of masses within the groups was normally distributed. Subsequently, the treatments of the beetles in the laboratory and field experiments diverged, as detailed in the sections below (see *Laboratory Experiment* and *Field Experiment* sections).

### Laboratory Experiment

Before starting the laboratory experiment, we kept the beetles in FH-1300 HiPoint (Taiwan) growing chambers at steady 15°C in the 12-h light and 12-h dark photoperiod and fed them 2–4 times per week with fresh spearmint leaves (*Mentha spicata*). After weighing, we moved each beetle into individual 120 ml jars (Laplex) filled with moist mixed soil (Kekkilä kaaliseos W HS R8019), approximately 75% of the volume of the jar. Then, we transferred the beetles gradually to a lower temperature with food, first from 15 °C to 10 °C and 12 h photoperiod for 2 wk. Thereafter, we moved them from 10 °C to 7 °C without light and food.

We started the experiment by dividing the beetles into 3 treatment groups: (i) steady temperature of 5 °C as control (5 °C; *N* = 50), (ii) steady temperature of 7 °C as treatment with higher temperature (7 °C; *N* = 48), and (iii) fluctuating 7 ± 3 °C as simulation of snowless winter (7 ± 3 °C; *N* = 46). In the 7 ± 3 °C group, the temperature alternated between 4 °C and 10 °C, each maintained for 10 hours, with transitional period of 2 h at 7 °C in between these temperatures (e.g., 10 h at 4 °C, then 2 h at 7 °C, then 10 h at 10 °C, then 2 h in 7 °C, and so on). The duration of the treatments was 5 months during the winter of 2021 in FH-1300 HiPoint (Taiwan) growing chambers. To maintain the optimal moisture during the experiment, we added 25 ml of water to the jars 1–2 times per month. Temperatures in the laboratory study are likely higher than typical field conditions. This is because they represent the lowest settings reliably manageable by the chambers used in the study.

After the treatment period of 143 days, we checked and recorded active individuals that were on top of the soil. We weighed the beetles and determined their mortality. Some of the beetles were weak or passive and were determined to be alive, while only the completely immobile ones were determined to be dead. After determining the survival and body mass, the beetles were frozen at −20 °C for further lipid analysis.

We measured the water/lipid/dry content (mg) of the viable beetles using the modified Folch method (Folch et al. 1957, Lehmann et al. 2012). Dead beetles were removed from the mass and the dry/water/lipid content analysis. First, the beetles were weighed using Mettler Toledo XS105 (Switzerland) analytical scale for fresh weight (mg). Thereafter, we dried the beetles at 55 °C in the oven for 72 h, after which we reweighed to determine their water content (fresh weight − dry weight; mg). Next, we transferred the dried beetles into 2:1 chloroform:methanol solution for 72 h for extraction of structural lipids, carbohydrates, glycerol, amino acids, and neutral lipids (Newman et al. 1972). Finally, we redried the beetles at 55 °C for 72 h and reweighed to determine their dry mass (mg). The method may overestimate the lipid content since it also removes other substances in addition to lipids (Williams et al. 2011). Consequently, our results should be interpreted as estimates of lipid-related body composition, intended to highlight differences between the experimental groups rather than precise quantifications of lipid mass.

We performed data analysis using the IBM SPSS Statistics program (version 28.0.1.1 (15)). Chi-squared test was applied to analyze the effects of treatment and sex on mortality. To investigate both the main effects and the interaction effect of treatment and sex on mass change percentages of the surviving beetles over time, we employed a 2-way analysis of variance (ANOVA) for the mass change percentages. The mass change percentages were calculated by dividing the start and end mass difference by the start mass and multiplied by 100. We included the treatment (different temperature conditions to which beetles were exposed), sex (male vs. female), and their interaction as main effects. We analyzed the effects of different temperature treatments on the water and lipid mass using 2-way analysis of covariance (ANCOVA). We set the treatment, sex, and their interaction as the factors and dry mass as the covariate. Where significant effects were found, we used the Tukey HSD post hoc test for pairwise comparisons, allowing a detailed interpretation of both the main and interaction effects. Dead beetles were excluded from the mass, water, lipid, and dry content analysis.

### Field Experiment

Before the field experiment, we kept the beetles in jars outdoors under a protective shelter and fed them 2–4 times per week with spearmint leaves (*Mentha spicata*). After weighing, we moved the beetles to the treatment block boxes filled with clay-rich soil from the beetle collection spot in arable land of Kortesuo-Viitaniemi, Jyväskylä, Finland (N 62.249188; E 25.727169). An extra 5 cm of air space was left between the soil surface and rim of the box, and filled with spearmint (*Mentha spicata*) plants and litterfall. The boxes were made from transparent Smart Store Classic 13 boxes (28 × 28 × 32 cm), an iron mesh added to the sides to allow temperature and water flow.

Beetles were divided into 2 treatments: (i) Snow removal and (ii) control. Each treatment had 2 blocks of 30 beetles (total *n* = 60). Temperatures were measured with 5 HOBO MX1220 (Onset Computer Corporation, USA) temperature data loggers, one in each block and one to record the air temperature. The loggers were placed on ground level and under the leaves to monitor the temperature in the area where beetles were expected to overwinter. The boxes, filled with soil and additional space for leaves and plant material, were dug into the arable land in Kortesuo-Viitaniemi. Before installing the boxes, we built a land drain from gravel under the boxes to prevent flooding. The boxes were installed so that the soil level inside each box was aligned with the surrounding ground surface while the rim and the lid remained approximately 5 cm below ground.

The snow was removed from the top of the snow removal treatment boxes within 1–7 days after the snowfall, while the snow in the control group was left undisturbed. During the snow removal events, we measured the snow depth using a 1-m-long metallic measuring stick, inserting it into the snow at 3 distinct, untouched locations around the area to calculate the average depth. We allowed the beetles to overwinter in these conditions for 6 months of which the continuous snowy period was 3.5 months. At the end of the treatment, we lifted the boxes from the ground and dug out the beetles from the soil, determined their mortality, and weighed their body mass. Each block represented a distinct experimental group with 30 beetles, and treatment referred to snow removal of control groups.

We analyzed the effects of treatment, sex, and treatment block on mortality using the chi-squared test. We analyzed the after-winter body mass differences from viable beetles using ANCOVA, with treatment and sex as factors and treatment block as a covariate.

## Results

### Laboratory Experiment

The overall winter mortality was low (<20%) in all the treatment groups, being higher in 7 °C and 7 ± 3 °C than in the 5 °C group (Table 1). All in all, increasing temperature also increased mortality (*X*^2^ = 8.92, *df* = 2, *P* = 0.012), but the fluctuation did not affect mortality (difference between 7 °C and 7 ± 3°C: *X*^2^ = 1.34, *df* = 1, *P* = 0.247).

**Fig. 1.**
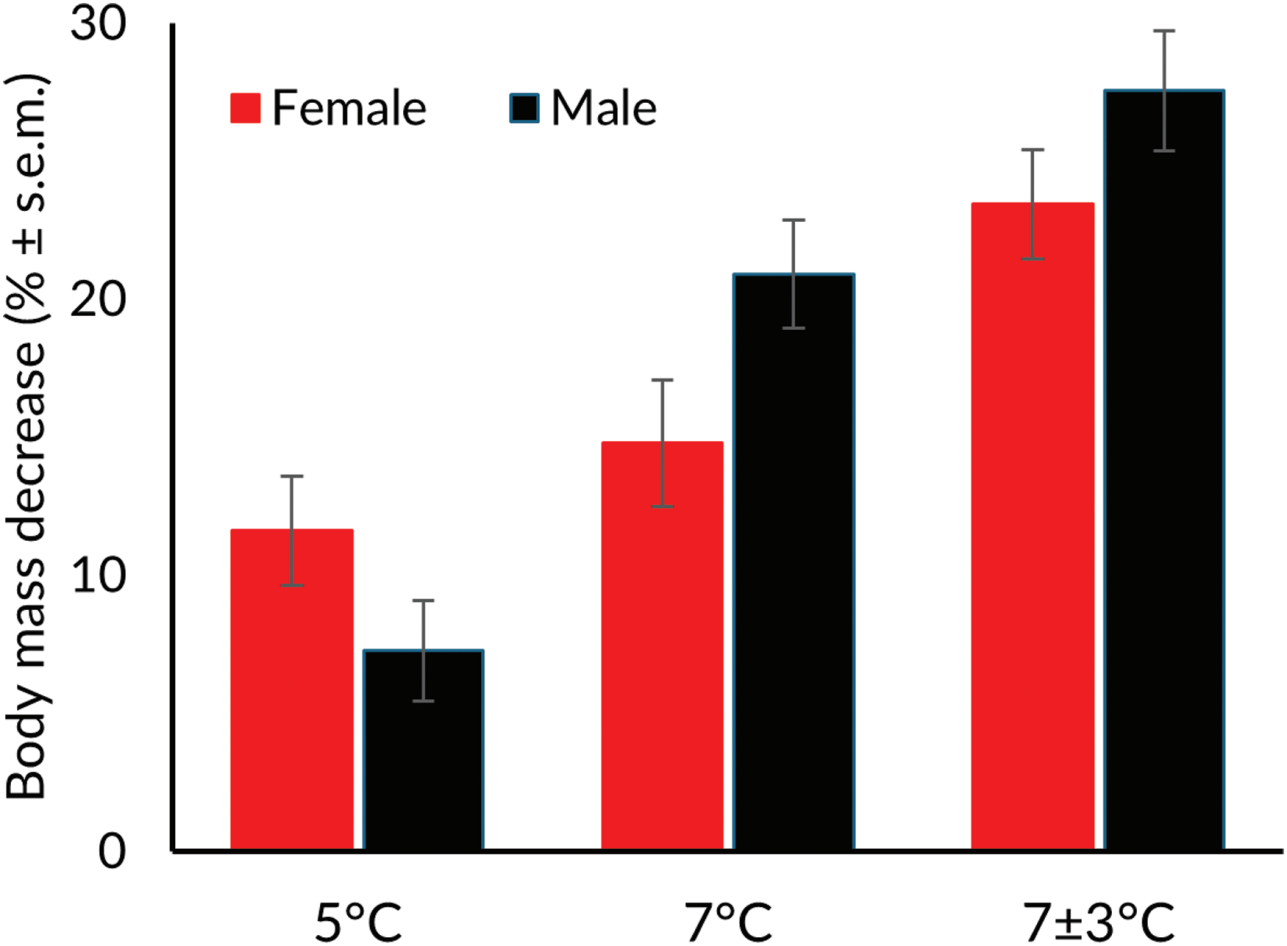
The mean body mass decrease (% ± s.e.m.) of female and male *Chrysolina polita* beetles in the 5 °C, 7 °C, and 7 ±* *3 °C groups.

Treatment (ANOVA *F*_2,1268_ = 32.7108, *P* < 0.001; Fig. 1) and the treatment sex interaction (*F*_1,126_ = 3.93, *P* = 0.022; Fig. 1) negatively affected body mass, while their sex alone (*F*_1,126_ = 1.39, *P* = 0.240) had no effect. Body mass decreased significantly in the 7 °C, showing an 8.4% reduction compared to the 5 °C group (Tukey: diff = −9.04, *P* < 0.001), while in the 7 ± 3 °C group, the reduction was even more pronounced at 16.1% (Tukey: diff = −16.04, *P* < 0.001). Additionally, the body mass decreased by 7.6% in the 7 ± 3 °C compared to the 7 °C (Tukey: diff = −6.99, *P* = 0.003). Besides the effects of temperature, there was a temperature–sex interaction; in the 5 °C group, females lost more body mass than males, while in the 7 °C and 7 ± 3 °C groups, the opposite occurred, with males losing more body mass.

**Fig. 2.**
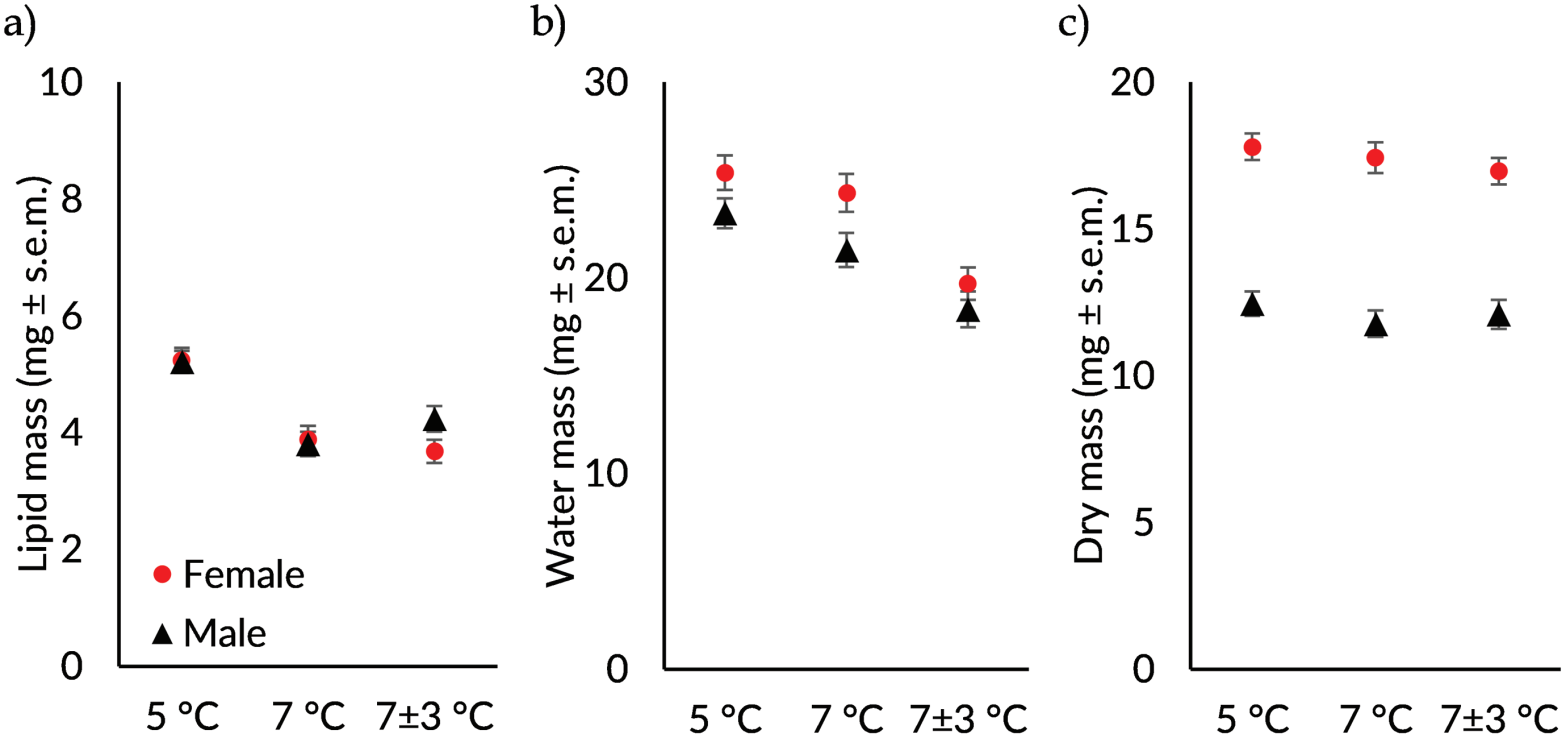
The estimated marginal means (mg, ± s.e.m.) of A) lipid, B) water, and C) dry mass of female (circle) and male (triangle) *Chrysolina polita* in the 3 treatment groups of 5 °C, 7 °C, and 7 ± 3 °C. The estimated marginal means for lipid and water mass are calculated using dry mass as a covariate.

Lipid mass was affected by treatment (ANCOVA *F*_1,125_ = 35.04, *P* < 0.001; Fig. 2A), whereas neither sex (ANCOVA F_1,125_ = 0.38, *P* = 0.540) nor sex and treatment interaction (ANCOVA *F*_2,125_ = 1.72, *P* = 0.183) affected lipid mass. Compared to the 5 °C group, the mean lipid mass was 1.4 mg lower in 7 °C (Pairwise comparisons: diff = 1.38, *P* < 0.001) and 1.3 mg lower in 7 ± 3 °C (Pairwise comp: diff = 1.27, *P* < 0.001) group. However, the 0.1 mg difference in mean lipid mass between 7 °C and 7 ± 3 °C groups was not significant (Pairwise comp: diff = −0.12, *P* = 0.556). As elucidated earlier, the lipid mass data aligns with the findings regarding mortality and body mass.

The water mass was also affected by the treatment (ANCOVA *F*_2,125_ = 24.77, *P* < 0.001, Fig. 2B) and sex (ANCOVA *F*_1,125_ = 4.28, *P* = 0.041). Similarly, the sex and treatment interaction did not show a significant effect on the water mass (ANCOVA *F*_2,125_ = 0.49, *P* = 0.614). In the 7 ± 3 °C group, the mean water mass was 5.7 mg lower compared to that of 5 °C (Pairwise comp: diff = 6.68, *df* = 128, *P* < 0.001), and 2.8 mg lower compared to the 7 ± 3°C (Pairwise comp: diff = 4.56, *df* = 128, *P* < 0.001) group. However, the difference of 2.9 mg in water mass between the 7 °C and 7 ± 3 °C groups did not reach statistical significance (Pairwise comp: diff = 1.82, *df* = 128, *P* = 0.072) and, thus, should be interpreted with caution. As shown in Fig. 2B, the females lost more water than males.

Finally, as expected, the dry mass was similar between the different treatment groups (ANOVA: *F*_*1*,126_ = 1.06, *P* = 0.350). Also, there was no treatment and sex interaction effect on dry mass (ANOVA: *F*_1,126_ = 0.33, *P* = 0.720). Females had on average 5.3 mg bigger dry mass than males (ANOVA: *F*_1,126_ = 193.5, *P* < 0.001; Fig. 2C).

In both 7 °C and 7 ± 3 °C groups, 90 % and 89 % of all the beetles were on the surface of the soil at the end of the experiment, which was significantly more than 24 % in the 5 °C group (Wald *χ*^2^ = 47.9, *df* = 2, *P* < 0.001). Furthermore, 84.6 % of the beetles that died during the experiment were found on the surface of the soil, exclusively within the 7 °C and 7 ± 3 °C groups, with no deaths reported in the 5 °C group (Table 1).

### Field Experiment

**Fig. 3.**
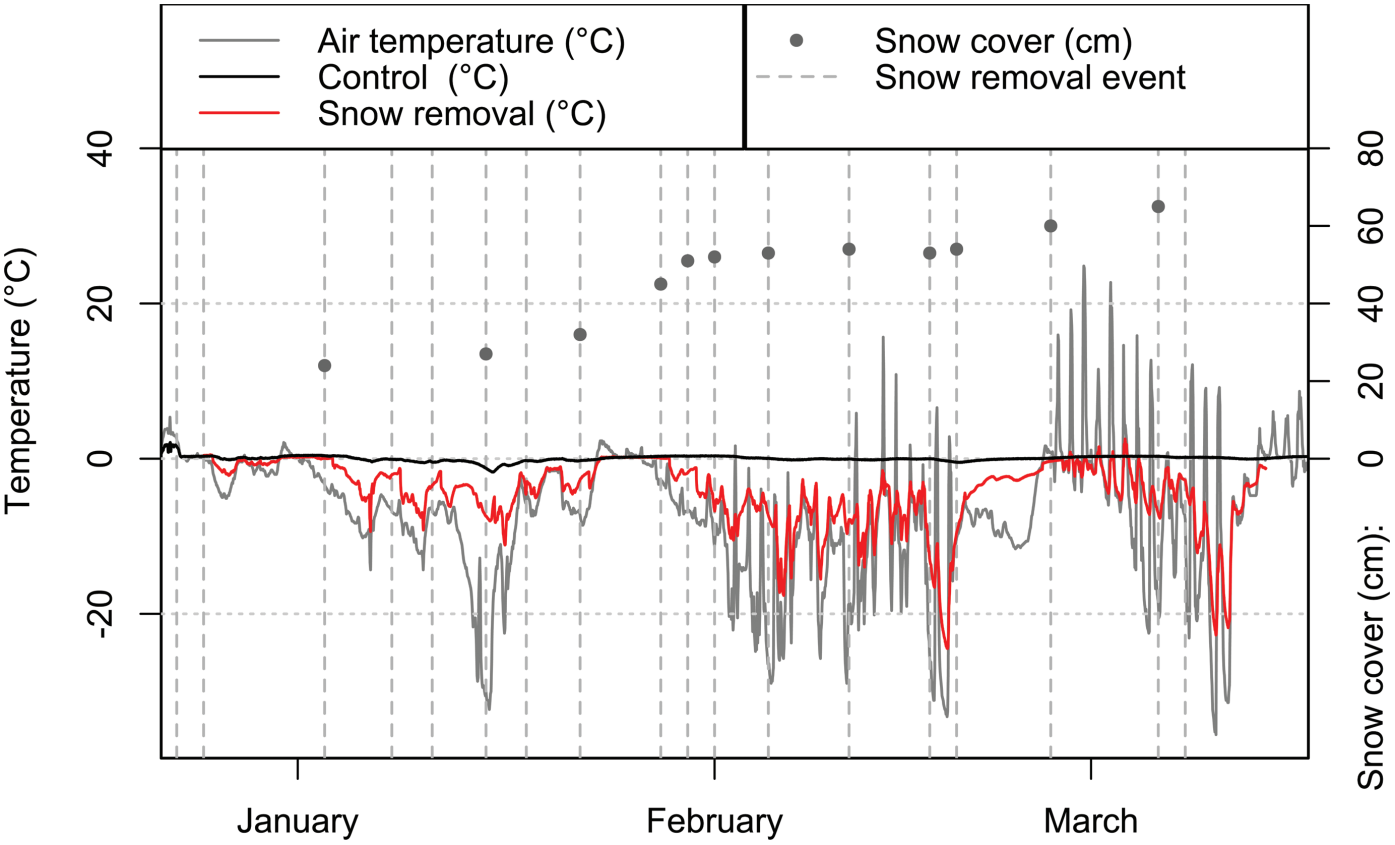
Variations in winter temperature (°C): The average winter temperatures measured at the soil surface, below the litterfall, in both the snow removal and control groups, based on 2 measurements each. The air temperature was recorded 1 m above the soil, centered among the 4 boxes, during the winter of 2021. The snow removal events are indicated by vertical dashed lines, and the snow cover, marked with dots, was estimated with a measuring stick.

The temperature data clearly shows the buffering effects of snow (Fig. 3). The snow removal treatment displayed stronger and more rapid fluctuation with lower mean temperature on ground level (−3.0 ± 5.0 °C, range −26.4 to 2.5 °C) than the control treatment, which consistently stayed in the steady zero (0.1 ± 0.3 °C, range −1.7 to 0.5 °C) throughout the snow cover duration. As anticipated, the temperatures recorded in the field experiment were consistently lower than those in the laboratory, with a difference of 5–8°C below the laboratory temperatures. On the other hand, Fig. 3 shows that 10 cm and 60 cm of snow cover provide a similar buffering effect. This observation indicates that the thickness of a snow layer does not significantly impact its ability to buffer temperature fluctuations, suggesting that only a thin layer of snow is sufficient for this purpose.

Despite the apparent differences in temperature conditions in the field experiment, there were no observed differences in the mortality of the beetles between the treatment groups (*X*^2^ = 1.03, df = 1, *P* = 0.309) and different treatment blocks (*X*^2^ = 1.03, *df* = 1, *P* = 0.309; Table 2). Mortality was low in both snow removal, and only males exhibited likely higher mortality compared to females (*X*^2^ = 3.39, *df* = 1, *P* = 0.066; Table 2). Subsequently, we found no differences in the postwinter mass between the treatments (ANCOVA *F*_1,112_ = 0.50, *P* = 0.481) and treatments sex interaction (ANOVA *F*_1,112_ = 0.13, *P* = 0.719). As expected, females were after winter on average 20.3 mg heavier than males (ANOVA *F*_1,112_ = 482.7, *P* < 0.001; Table 2).

## Discussion

Climate change is predicted to increase mean air temperature and decrease snow cover in temperate zone (IPCC 2021, Ruosteenoja and Jylhä 2021). These changes in winter conditions can impact the ability of many insect species to successfully overwinter. We tested how increases in both overwintering temperature and temperature variation in the laboratory as well as snow removal in the field affect the knotgrass leaf beetle, *Chrysolina polita*. We found that snow removal decreased soil temperatures and increased temperature fluctuations in the field but these, in turn, had no effect on the winter survival of the beetles. However, temperature increase with and without fluctuations increased mortality in the laboratory although it was relatively low in all of the treatment groups. These results indicate that *C. polita* can tolerate changes towards colder overwintering temperatures better than towards warmer overwintering temperatures. Similarly, the beetles tolerated the increase in fluctuating cold temperatures better than the fluctuating warm overwintering temperatures, suggesting that it is a hardy species that seems to be well adapted to the current winter temperature conditions of the temperate zone.

Climate change is expected to lead to thinner snow cover, potentially affecting soil temperatures. Our data shows that snow removal decreased the mean winter temperatures from 0 °C under the snow cover to −3 °C without it. However, despite this reduction, snow removal did not impact beetle mortality or result in significant changes in body mass when compared to the control treatment. This result aligns with prior field studies with similar research setup involving a willow leaf beetle (*Chrysomela aeneicollis*; Roberts et al. 2021) and deer ticks (*Ixodes scapularis*; Burtis et al. 2016). Since *C. polita* is known to overwinter under leaves and not to burrow extensively in the soil (Lehmann et al. 2015) our inference is that similar mortalities between the field treatments are unlikely to be attributed to variations in the beetles’ choice of soil layers for temperature regulation.

Contrary to the field conditions, what was clear from our laboratory experiments was that if we increased the overwintering soil temperature (+2 °C, from 5 °C to 7 °C), the beetles increased lipid consumption resulting in a lower body mass and higher mortality compared to the control group (see Fig. 1). This result of elevating energy stress in higher temperatures is also in line with the previous findings made with other insect species overwintering as an adult, e.g., *C. aeneicollis* (Roberts et al. 2021), boreal wood ant (*Formica aquilonia*; Sorvari et al. 2011) and bumblebee (*Bombus lucorum*) queens (Vesterlund et al. 2014). These results together suggest that *C. polita* can tolerate changes towards colder overwintering temperatures better than towards warmer temperatures. Our results underline the importance of understanding future winter temperature changes and testing the scenarios also in the field.

The interactive effect between treatment and sex suggests that females may be more susceptible to higher winter temperatures compared to males. This increased sensitivity could likely be attributed to females having greater dry mass (Fig. 2C) despite similar lipid masses (Fig. 2A) between the sexes. This could heighten their metabolic demands, thus generating more body heat. Additionally, their larger size can make it more difficult for them to release this excess heat effectively and, thus, increase their resistance to rapid temperature changes.

Higher mortality in the laboratory conditions could arise if the increase in the overwintering temperatures can also change the behavior of the individuals. There were some suggestions for this, as in the laboratory experiment, more beetles on the higher temperature treatments were on the top of the soil at the end of the experiment. This indicates that the beetles in the higher mean temperatures might break the diapause earlier and climb to the soil surface. This could partially account for the increased lipid consumption in the higher temperature treatment if the elevated temperatures prompt the beetles to emerge from the soil and search for food. If beetles break the diapause before the adverse season is over, this might be detrimental. However, there are suggestions that if the winter temperatures increase, the duration of thermal winter will also shorten (Ruosteenoja et al. 2019). Thus, the outcome of winter changes might then depend on the matched changes in the phenology of the host plants of these herbivores.

The limitation of this study was the lack of humidity measurements, which are likely important for the overwintering success (see Rull et al. 2019, Musiolek et al. 2023, Klockmann and Fischer 2019, Sauer et al. 2022). In the laboratory, beetles exposed to fluctuating temperatures lost more body mass (Fig. 1), particularly water mass (Fig. 2B), possibly due to soil desiccation. Despite monthly watering, the soil in these jars appeared dryer than in the control. This moderate effect observed in the laboratory experiment may be more attributable to the desiccation of the soil rather than temperature fluctuations alone. Moreover, mortality rates were consistent across groups with the same mean temperature (7 °C), despite fluctuation, suggesting that changes in water mass composition do not influence mortality as significantly as losses in lipid mass do.

Contrary to previous studies indicating a requirement of 20 cm snow depth for the temperature buffering effect (Goncharova et al. 2019), we observed that even a thin layer of snow was enough to effectively buffer the air temperature (see Fig. 3). This might mean that the overwintering conditions at the temperate zones, with predicted increased precipitation and snowfall, could somehow balance the adverse effects of the increase in the temperature. However, although we occasionally measured the snow depth, we did not specifically investigate snow depth effects on temperature buffering. Therefore, our data is insufficient to reliably test this hypothesis, and further research is necessary to draw definitive conclusions.

In addition, while climate change can cause more unexpected events and rapidly changing conditions which makes the consequences hard to predict, more research is also needed to better understand the physiological mechanisms climate change has on the overwintering survival of insects. Future research topics should also include research on the transgenerational effects of the changed overwintering conditions. For instance, the influence of the increase in the melting-freezing cycle on reproduction and progeny and its potential direct impact on future generations could be a prospective avenue for further research.

In conclusion, our study demonstrated that *C. polita* exhibits greater tolerance to lower temperatures, with field observations confirming low mortality rates even under extremely cold conditions. Conversely, laboratory results showed a low mortality rate at 5 °C that increased significantly at 7 °C, suggesting that the survival threshold of *C. polita* may lie within a narrow temperature range where even slight increases can impact survival. Additionally, it is crucial to consider the potential impact of humidity on survival—a factor not measured in this study but essential for understanding the comprehensive ecological dynamics of winter survival. These insights not only deepen our understanding of the thermal resilience of *C. polita* but also highlight the intricate interplay of environmental factors in determining the winter survival of temperate insect species amid a changing climate.

## Data Availability

Data will be made available at Jyväskylä University Digital Repository website at https://jyx.jyu.fi/.
